# Neurological manifestations in dogs with acute leukemia

**DOI:** 10.3389/fvets.2024.1385093

**Published:** 2024-07-18

**Authors:** Filipa L. S. Lyseight, Charles Pittaway, Ruth Dennis, Giunio B. Cherubini

**Affiliations:** ^1^Oncology Service, Dick White Referrals, Part of Linnaeus Veterinary Limited, Cambridgeshire, United Kingdom; ^2^Diagnostic Imaging Service, Dick White Referrals, Part of Linnaeus Veterinary Limited, Cambridgeshire, United Kingdom; ^3^Neurology and Neurosurgery Service, Dick White Referrals, Part of Linnaeus Veterinary Limited, Cambridgeshire, United Kingdom; ^4^Department of Veterinary Sciences, University of Pisa, Pisa, Italy

**Keywords:** bone marrow magnetic resonance imaging, acute leukemia magnetic resonance imaging, veterinary neuro-oncology, dogs with acute leukemia, central nervous system leukaemia

## Abstract

Canine acute leukemia is a rare hematopoietic neoplasm. Neurological abnormalities have been frequently reported in dogs with acute leukemia (AL). However, the description of the presentation and findings are limited. This study aimed to describe the clinical findings in dogs with acute leukemia presenting with neurological signs as their primary complaint. The database of a private referral hospital was searched for cases that presented to the neurological department with neurological deficits and were subsequently diagnosed with acute leukemia. Six cases were included; all had neurological clinical signs and an abnormal neurological examination. All cases had a focal neuroanatomical localisation on neurological examination (brain *n* = 4; spinal = 2). Out of the four dogs with a complete magnetic resonance imaging (MRI) study, there was an ill-defined infiltrative pattern with abnormal signal intensity in the central nervous system (CNS) in three dogs and the loss of grey and white matter differentiation in the brain (*n* = 2) and/or spinal cord (*n* = 2). Other MRI findings included abnormal meningeal enhancement (*n* = 3), changes affecting spinal nerves and epaxial muscles (*n* = 2), and lymphadenopathy in the field of view (*n* = 2). The bone marrow assessment on MRI showed evidence of signal change (*n* = 3), characterized by a loss of normal fat opacity and an abnormal degree of contrast enhancement. The cerebrospinal fluid (CSF) analysis of the four dogs showed an increased protein level with non-specific pleocytosis and without evidence of malignant cells. Treatment with cytotoxic medication was implemented in two dogs. The dogs diagnosed with acute leukemia had focal neuroanatomical localisation, an infiltrative CNS pattern, and bone marrow remodulation on MRI with an increase in CSF protein and negative cytology analysis.

## 1 Introduction

Hematopoietic tumors can originate from the cells of the immune system (e.g., lymphoma) or in the bone marrow (e.g., leukemia). Acute leukemia (AL) is defined as the uncontrolled proliferation of immature (blast) lymphoid or myeloid cells in the bone marrow, which can be classified as acute lymphoid leukemia (ALL) or acute myeloid leukemia (AML), respectively (1–3). AL occurs when there is a rapid proliferation of blasts in the early stage of development, replacing the normal hematopoietic cells, which can disseminate in the blood and/or infiltrate organs such as the lymph nodes, liver, and spleen (4).^.^ The World Health Organization (WHO) defines acute leukemia as an increased presence of blasts in the bone marrow (>20% for AML and >25% for ALL) or peripheral blood (> 20% for AML and ALL) (5). In chronic leukemia (CL), there is an accumulation of maturing and mature cells in the bone marrow. Chronic leukemia also has a chronic onset of symptoms, contrary to acute leukemia, which has a rapid clinical course (4, 6).

Dogs with AL commonly present with non-specific signs such as lethargy and inappetence. The physical examination findings include pyrexia, mild peripheral lymphadenopathy, abdominal organomegaly, abnormal neurological examination, pale mucous membranes, and hemorrhage (e.g., epistaxis). The most common haematological finding is a high number of circulating blasts and bicytopenia (over 50% of dogs), characterized by anemia and thrombocytopenia. A moderate increase in the level of alkaline phosphatase (ALP) is the most reported biochemical finding (1, 7–10). Approximately 30% of dogs have a cranial-thoracic mass identified on thoracic radiographs. The most common findings in the ultrasound of the abdomen are splenomegaly, hepatomegaly, and intra-abdominal lymphadenopathy (9, 11). Leukemia is diagnosed in the clinic by cytology analysis (morphological and cytochemical analyses) of peripheral blood, bone marrow, and/or infiltrated organs (1, 3, 12). In recent times, flow cytometry has improved the diagnosis (CD34-positive) and subtyping of leukemia (3, 9, 12). The survival rates are poor without the use of any cytotoxic medication (less than 5 days). Dogs treated with multiagent chemotherapy protocols also have a poor prognosis, with median survival rates reported varying from 9 to 120 days (9, 12).

Neurological clinical signs have been frequently reported in dogs with acute leukemia; however, the description of clinical findings is limited (8–10). A few case reports have documented neurological signs as the primary complaint (13–16). Ataxia, head tilt, head pressing, circling, acute blindness, mandibular paralysis, photophobia, seizures, and diffuse atrophy of the cranial skeletal muscles have been reported (10, 13–15). Spinal cord infiltration has been associated with tetraparesis, flaccid paraplegia, the absence of nociception and spinal reflexes, pain, and lower motor neuron incontinence (15–17). The description of diagnostic neuroimaging findings is limited to two cases. The first case showed bilateral, extradural compression from C2 to C5 in the myelogram; this was confirmed as a subtype of acute leukemia on necropsy (15). The second case showed infarcts and an infiltrative pattern within the right thalamic region of the brain on MRI, suspected to be AL (10). Cerebrospinal fluid cytology and biochemistry were reported in two dogs, showing a high number of blast cells (>130 per μL) and increased protein (12–15 mg/dL) (13, 17).

The MRI is the only imaging modality that allows direct visualization of the bone marrow (18). The bone marrow is composed of fat cells and active hematopoietic cells, which determine its signal intensity on MRI. A mature bone marrow appears hyperintense to skeletal muscle and intervertebral disc, and similar to subcutaneous fat with suppression of signal in Short Tau Inversion Recovery (STIR) or with spectral fat saturation (19–21). Haematological malignancies cause bone marrow replacement that can be seen as hypointensity on T1- and hyperintensity on T2-weighted images. This finding is non-specific and is also associated with inflammatory or metabolic disorders. The MRI of the bone marrow is sensitive for the diagnosis of lymphoma and leukemia in human medicine and is used to assess the response to treatment (18, 19, 22, 23).

This study aims to describe the clinical findings in dogs with acute leukemia, presenting with neurological signs as their primary complaint. The secondary aim was to characterize the changes in the bone marrow of MRI in dogs with acute leukemia.

## 2 Materials and methods

The medical records of dogs presenting between January 2010 and December 2022 from the Neurology Department of a single private referral center were retrospectively reviewed. The inclusion criteria were as follows: (1) having a neurological examination performed by a board-certified neurologist or resident and (2) being diagnosed with acute leukemia based on the cytology of bone marrow or peripheral blood (> 20% blast cells) (1, 9) or flow cytometry. Cases were excluded if there was clinical suspicion of multicentric stage V lymphoma (e.g., peripheral lymph nodes >1 cm) or if other causes of neurological complaints were identified. All magnetic resonance imaging studies (Hitachi Aperto Lucente 0.4 Tesla, Berkshire, UK) were reviewed by a European College of Veterinary Diagnostic Imaging (ECVDI) diplomate. The initial cytology and histopathology analysis reports were evaluated by a board-certified veterinary clinical pathologist on the initial presentation. The data collected included signalment (age, sex, neutering status, body weight, and breed), clinical history, clinical signs, general clinical examination, neurological examination, diagnostic procedure findings, treatments, and outcomes.

## 3 Results

A total of eight dogs met the inclusion criteria; two were excluded as one dog had an intervertebral disc extrusion on MRI and the other dog had stage V lymphoma. A total of six dogs were finally included. There were five males and one female, all neutered. The median age was 3.6 years (range 2.5–4.3), and the median body weight was 21.35 kg (range 3.8–38 kg). The The represented breeds breeds were English Springer Spaniel (*n* = 2), Border Collie, Chihuahua, Golden Retriever, and French Bulldog. All owners reported a history of acute (*n* = 1) or progressive clinical signs with acute deterioration (*n* = 5), including abnormal ambulation (intermittent unilateral thoracic limb lameness *n* = 2; ataxia *n* = 2; paraparesis *n* = 2) and intermittent pain (*n* = 2), which started from 12 h to 3 weeks before presentation. One dog exhibited additional non-specific clinical signs, including lethargy, intermittent panting, inappetence, and hypersalivation.

During neurological examination, all dogs showed changes including depressed mentation status (*n* = 3), paresis (paraparesis *n* = 3; hemiparesis *n* = 1), generalised ataxia (proprioceptive *n* = 3; vestibular *n* = 1), absence of menace (with normal palpebral reflex *n* = 1; with absence of palpebral reflex *n* = 1), rotatory nystagmus (*n* = 1), decreased facial and nasal sensation (*n* = 1), decreased to absent postural reactions (bilateral pelvic limbs *n* = 2; right-sided *n* = 1), decreased muscle tone and segmental spinal reflexes (bilateral thoracic limbs *n* = 1; bilateral pelvic limbs *n* = 2), low head carriage with plantigrade stance (*n* = 1), and pain (diffuse spinal pain *n* = 2; cervical spine pain *n* = 1). Neuroanatomical localisation was consistent brain (forebrain *n* = 2; vestibular apparatus *n* = 1; diffuse brain *n* = 1) and spine (cervical *n* = 1; diffuse myelopathy with predominance in L4-S3 *n* = 1). Based on general clinical examination, three dogs were detected with abnormalities including mild peripheral lymphadenopathy characterized by firm peripheral lymph nodes <1 cm (*n* = 2), pyrexia (*n* = 1), and generalized muscle atrophy (*n* = 1).

**Figure 1:**
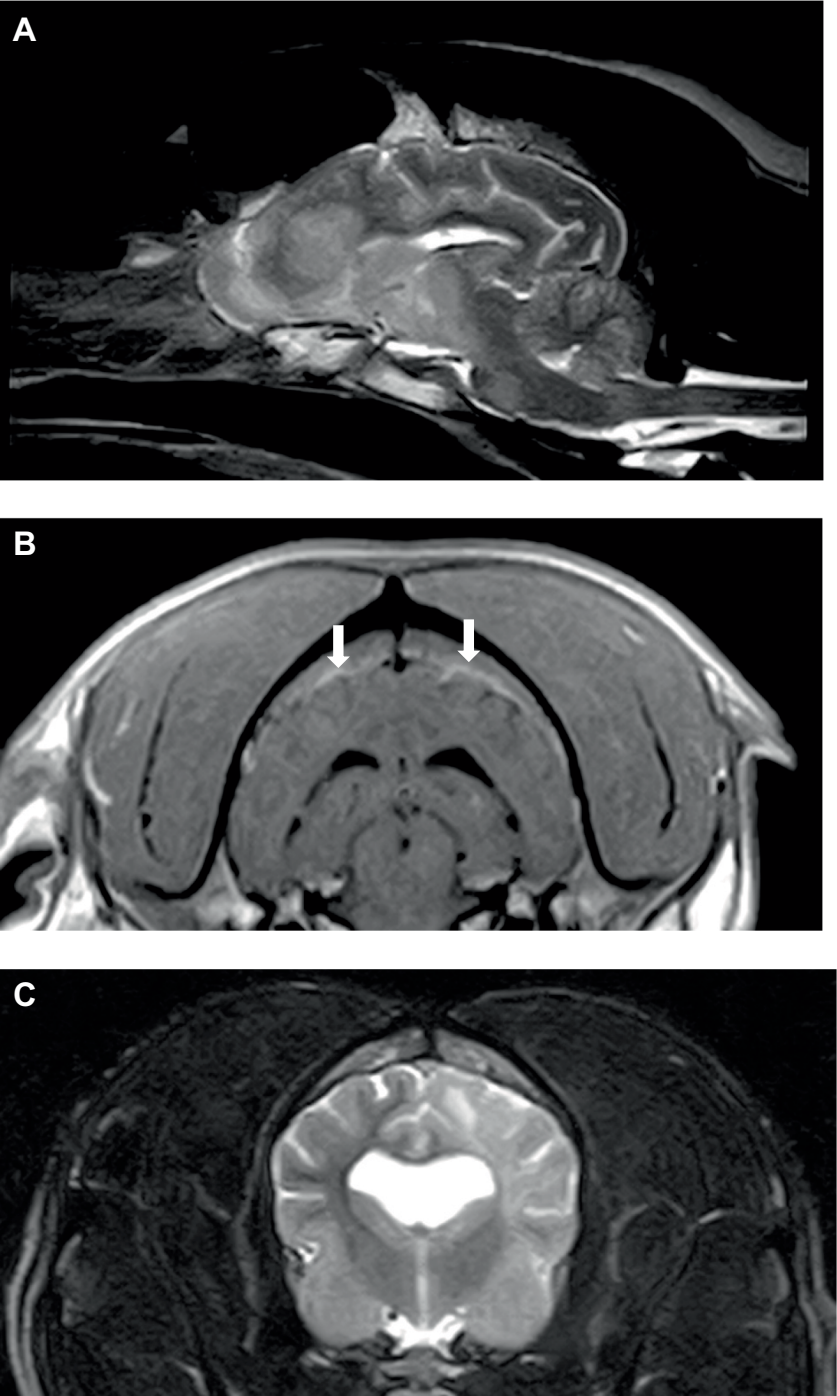
Sagittal T2W **(A)** and transverse post-contrast T1W **(B)** images of the brain of a 6-year-old English Springer Spaniel showing multifocal, ill-defined brain lesions causing minimal mass effect **(A)** and thickening and inflammation of pachymeninges **(B)** (arrowed). Transverse T2W **(C)** image of the brain in a 2-year-old English Bulldog showing diffuse left cerebral pathology with a mild mass effect.

Of the six dogs, three dogs underwent an MRI procedure of the brain and two dogs underwent an MRI procedure of the spine. One dog had the study interrupted as the diagnosis of acute leukemia was achieved via peripheral blood cytology. The obtained sagittal and transverse T2 weighted (T2W) images of the brain and surrounding bones appeared normal. There was lymphadenomegaly (mandibular and retropharyngeal) and enlargement of the right tonsil. The complete brain studies in the other two dogs included sagittal T2W, transverse T2W, FLAIR, T2*GE, and T1W, and post-contrast T1W in three planes; an additional dorsal oblique STIR and dorsal post-contrast T1W image were obtained in one dog. Both studies showed widespread/multifocal, ill-defined patches of hyperintensity on T2W and STIR in both the grey and white matter of the brain, which showed faint, ill-defined contrast enhancement increasing with time. In one dog (Figures 1A,B), these lesions were observed in all areas of the brain, and these lesions were associated with minimal mass effect, whereas, the second dog had diffuse, left-sided pathology only with mild to moderate mass effect (Figure 1C). There was an increased meningeal contrast enhancement (pachymeninges *n* = 2; leptomeninges *n* = 1), and the meninges were thickened in one dog. The cranial part of the spinal cord was included in one study and showed subtle, ill-defined patches (as described for the brain) at C1-C2. One dog had hyperintensity of the adjacent musculature.

**Figure 2:**
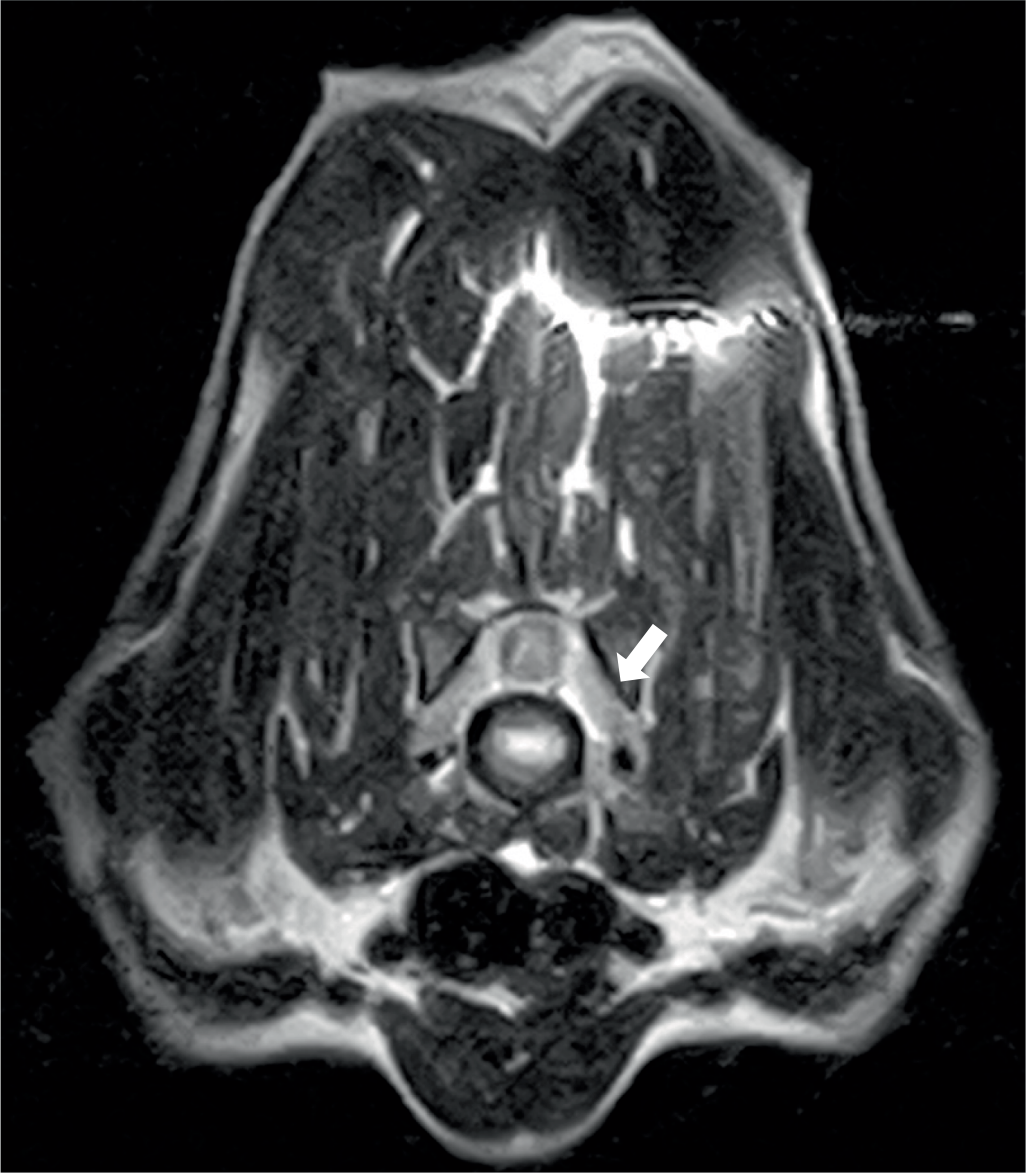
Transverse T2W image at the level of the C6-7 disc space in a 9-year-old Spaniel cross showing diffuse thickening of the C7 spinal nerves (arrowed) and/or surrounding soft tissues.

**Figure 3:**
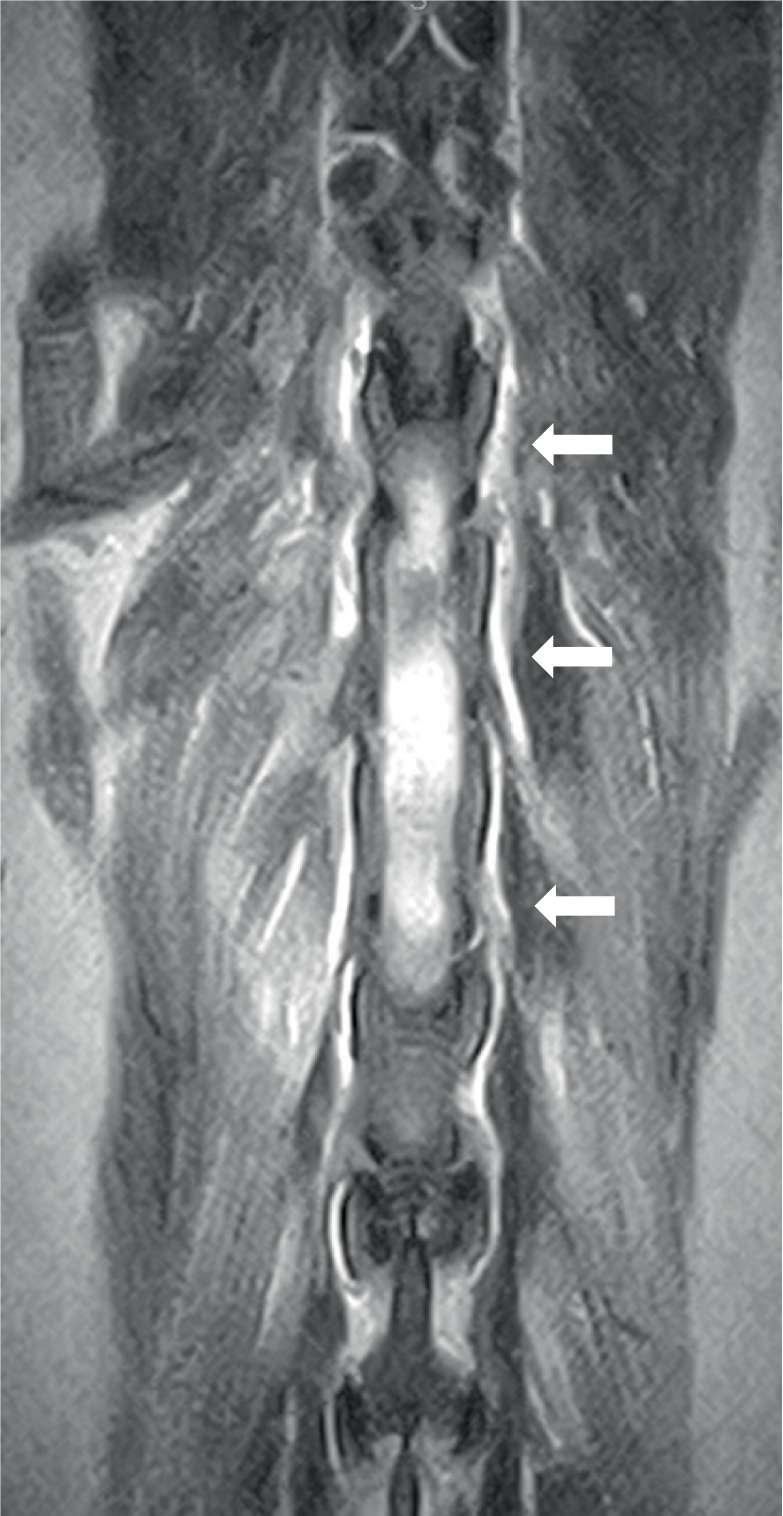
Dorsal T2W image of the thoracolumbar area in a 7-year-old golden retriever showing bilateral, diffuse, and epaxial muscle hyperintensity (arrowed).

Two dogs underwent spinal MRIs. The cervicothoracic spine study included sagittal T2W, STIR, pre- and post-contrast T1W, and transverse T2W and STIR images, which showed moderate, diffuse, spinal cord swelling of C3-C5 with subtle, patchy cord hyperintensity as observed in STIR. The thoracolumbar study included sagittal and dorsal T2W and sagittal and transverse STIR images, and in the same dog, a lumbosacral study included sagittal T2W, STIR, and T1W, and transverse and dorsal T2W. There were no abnormalities noted in the spinal cord at either site. Both the dogs had evidence of spinal nerve inflammation; in the first dog, this was bilateral and multifocal (Figure 2) and the other dog had a thickened spinal root at L1 within the vertebral canal. In both cases, the adjacent musculature (epaxial or longus colli) showed severe, bilateral, well-defined, asymmetric hyperintensity (Figure 3). One dog was considered to have an equivocal absence of meningitis, and the other had dispersed epidural changes suggestive of meningitis or empyema. One dog had splenomegaly.

**Figure 4:**
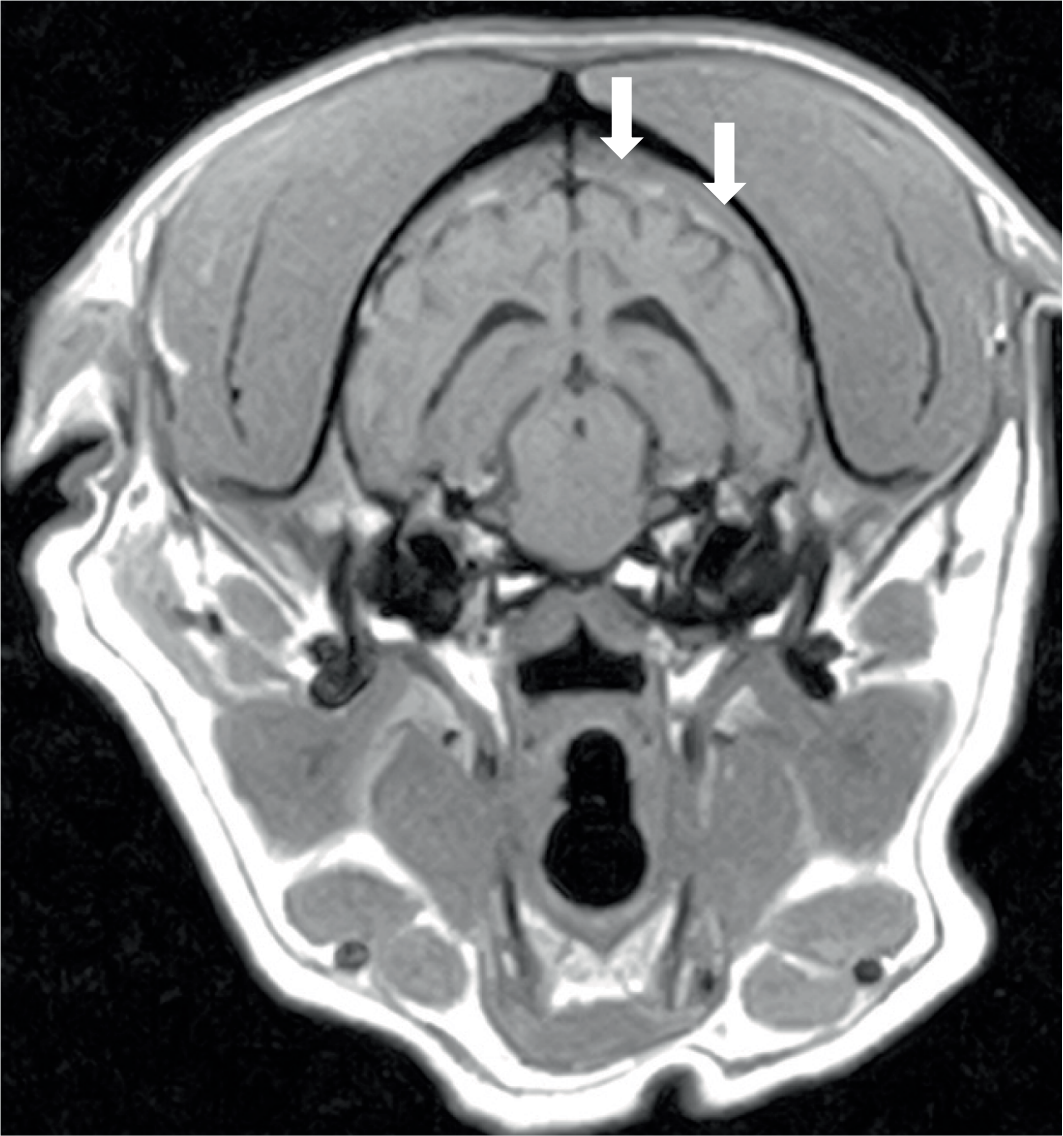
Transverse T1W image of the brain of a 6-year-old English Springer Spaniel showing a reduction in the normal hyperintense signal from calvarial bone marrow (arrowed). The same dog, as shown in Figures 1A,B.

**Figure 5:**
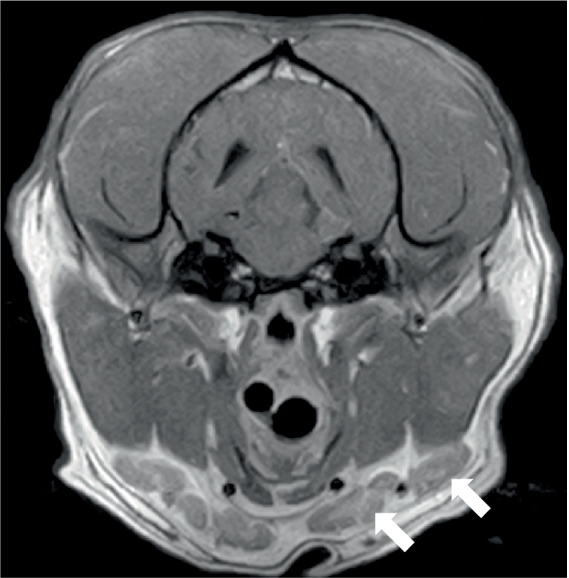
Transverse post-contrast T1W image of the head of a 2-year-old English Bulldog showing enlarged mandibular lymph nodes (arrowed). The same dog is shown in Figure 1C.

Three dogs showed bone marrow remodeling on MRI. This was characterized by heterogeneous loss of fat opacity of normal bone marrow on T1 and a lack of suppression on STIR (Figure 4), and one dog showed mildly increased contrast enhancement of the bone marrow. These changes were noted in bones adjacent to abnormal musculature or meninges, as described above (calvarial bone *n* = 2; manubrium and scapulae *n* = 1). All dogs had mild to moderate lymphadenopathy (Figure 5) of various lymph nodes within the field of view (FOV) (Figure 5).

Cerebrospinal fluid was obtained from the cisterna magna of four dogs for cytology and biochemistry analysis. None of the dogs had a neoplastic population on cytology (Tables 1, 2). One dog had an incisional biopsy of the longus colli muscle (hyperintense on MRI) for histopathology and culture. The histopathology analysis revealed mature adipose tissue and neutrophilic inflammation consisting of an accumulation of degenerate neutrophils. Aerobic and anaerobic enrichment cultures were negative. Additional tests were conducted on the same dog, including a vector-borne snap test; serology for *Leishmania, Toxoplasma* spp.*, and Neospora* spp.; and blood culture. All the results were negative.

**Table 1:** Most common abnormal findings on complete blood count (CBC), biochemistry (MBC), cerebrospinal fluid cytology, and biochemistry (HCT, haematocrit; NEU, neutrophils; PLT, platelets; ATC, atypical cells or circulating lymphocytes; ALP, alkaline phosphatase; ALB, albumin; GLB, globulin; CRP, c-reactive protein; NC, nucleated cells).

	Case	1	2	3	4	5	6		
Parameter								Unit	Ref.
CBC	HCT	0.32	0.32	0.24	0.30	0.31	0.29	L/L	0.37–0.55
	NEU	1.19	4.5	32.65	2.12	1.44	11.61	x 10^9^/L	3.0–11.5
	PLT	50	0	56	72	0	78	x 10^9^/L	200–500
	ATC	115.16	758	251.2	39.50	24.93	17.42	x 10^9^/L	0 0–0.01
MBC	ALP	1,505	117	1,687	558	831	1,372	IU/L	17–86
	GLB	46	23	45	68	39	22	g/L	24–38
	ALB	26	24	23	22	30	22	g/L	25–38
	CRP				47		106	mg/L	< 10
CSF	NC	11			<1	27	9	U/L	0–5
	CSF protein	0.74			0.39	2.09	1.06	g/L	0.15–0.30
	CSF lactate					2.89		mmol/L	1.2–2.0

**Table 2:** Description of cytology analysis of peripheral blood, cerebrospinal fluid, flow cytometry from peripheral blood, and bone marrow findings.

	Blood film cytology	CSF cytology	Flow cytometry	Bone marrow cytology	Final Diagnosis
1	Medium-large mononuclear cells with medium-large, round nuclei with dispersed chromatin and occasionally small nucleoli. Basophilic, scant cytoplasm. Low numbers of mitoses are observed.	Lymphocytic pleocytosis	CD79+, CD45+,CD34+, CD3-, CD4-, CD8-, CD5-, CD21-, MUM-1-, CD14-, MHC II-		Acute leukemia, B-cell lineage
2	High numbers of immature cells with a high N:C ratio, round nuclei with dispersed chromatin, and occasionally small nucleoli. The cytoplasm was scant to moderate and moderately basophilic scant cytoplasm. Other cells had lobulated nuclei and a large amount of cytoplasm, with a monocytic appearance.				Acute leukemia, suggestive of myeloid origin
3	High numbers of immature cells with a high N:C ratio, round nuclei with dispersed chromatin, and occasionally small nucleoli. The cytoplasm was scant to moderate and moderately basophilic scant cytoplasm. Other cells had lobulated nuclei and a large amount of cytoplasm, with a monocytic appearance.				Acute leukemia, suggestive of myeloid origin
4	Small to intermediate lymphocytes with mild to moderate basophilic cytoplasm; round, indented nuclei. Smaller forms have condensed chromatin, whereas larger forms have finely granular chromatin with 0–2 small, mostly indistinct nucleoli.	There was no evidence of pleocytosis. There were monocytoid cells, macrophage-like with plump oval or round, paracentral nuclei. Moderate to abundant pale basophilic cytoplasm with low to moderate numbers of small to moderate-sized discrete clear vacuoles.		The monomorphic population consists of intermediate to large mononuclear cells with medium to large, round, eccentrically positioned nuclei that contain finely stippled chromatin and rarely 1–3 variably distinct nucleoli; small quantity of mild basophilic cytoplasm. The mitotic rate is 7–8/5 HPF 60x.	Acute leukemia (hematopoietic lineages) could not be assessed given the lack of spicules
5	Intermediate lymphocytes with round or indented nuclei, rarely complex, with finely granular to coarsely stippled chromatin and rare nucleoli. Scant to low amounts of mid/deep basophilic cytoplasm.	Mixed, predominantly mononuclear pleocytosis (66% lymphocytes, 11% large mononuclear cells, 21% neutrophils, and 2% eosinophils).	CD79a+ (weak), MHCII (weak), CD45+, CD34+, CD3-, CD5-, CD4-, CD8-, MUM-1-, CD14-, MHC II-		Acute leukemia, suggestive of B-cell origin
6	Small to intermediate lymphocytes, scant, moderately to deeply basophilic cytoplasm, and round, rarely bilobed nuclei. Small cells with mature chromatin. Intermediate cells with more open chromatin and occasional nucleoli.	Mild mixed, predominantly mononuclear pleocytosis. Nucleated cells were predominantly monocytoid cells and small lymphocytes with rare neutrophils. Monocytoid cells were macrophage-like with plump oval or round, paracentral, nuclei. Abundant and vacuolated cytoplasm.	CD3+; CD5+, MHCII+ (weak), CD45+, CD34-, CD79a-, CD4-, CD8-, CD5-, CD21-, MUM-1-, CD14-, MHC II-		English bulldog T-cell leukemia

The hematology and blood film analysis results on presentation were available for all dogs. All dogs with leucocytosis and cytology analysis of peripheral blood were considered diagnostic of leukemia in four out of six dogs. All dogs had severe thrombocytopenia and mild non-regenerative anemia, a median platelet count of 53 × 10^9^/L (range 0–78, R.I.: 200–500), and a median haematocrit of 0.30 L/L (range 24–32, R.I.: 0.37–0.55). Three dogs had pancytopenia with mild neutropenia (Table 1). In the peripheral blood biochemistry, all dogs had an increased alkaline phosphatase with a median of 1,102 U/L (range 117–1,687, R.I.: 17–86). Mild to moderate hyperglobulinemia was identified in four dogs, and c-reactive protein was increased in the two dogs in which it was measured (Table 1). Additional tests were conducted on four dogs for the diagnosis of acute leukemia including flow cytometry (*n* = 3) and bone marrow cytology and biopsy (*n* = 1) (Table 2).

Four dogs were humanely euthanized upon the diagnosis of acute leukemia. One dog received dexamethasone (0.1 mg/kg PO q24 hours), lomustine (50 mg/m^2^ PO), levetiracetam (20 mg/kg q8 hours), and prophylactic amoxicillin/clavulanic acid (20 mg/kg PO q12 hours) due to neutropenia. A transient improvement was reported by the owner, but the patient deteriorated and was euthanised after 4 days. The second dog received dexamethasone (0.1 mg/kg PO q24 hours) and cytarabine arabinoside (210 mg/m^2^ SC). Prior to the discharge, proprioception and gait had returned to normal. After 10 days, the patient was reported to have a good quality of life, and the neurological examination was within normal limits except for mild, persistent, unilateral thoracic limb lameness. However, there was a progression of circulating atypical cells, but the owner declined a multiagent chemotherapy protocol. A second dose of cytarabine arabinoside was administered, and glucocorticoid (dexamethasone) therapy was maintained as above. The patient was humanely euthanized at 4 weeks after diagnosis (7 days after the second cytarabine treatment) with a history of anorexia, vomiting, and Pelvic limb swelling.

## 4 Discussion

This study aimed to describe the neurological presentation and clinical findings of dogs with acute leukemia. Six young adult dogs presented with neurological signs associated with focal neurolocalization. Abnormal ambulation and posture were the most common findings. All had thrombocytopenia, mild anemia, and increased alkaline phosphatase. However, based on peripheral blood film analysis, only four dogs were diagnosed with acute leukemia. On MRI, five dogs had lymphadenopathy, and three had hyperintense patches (T2W and STIR) in the spinal cord and/or the brain associated with meningeal enhancement and bone marrow remodeling. Increased protein was noted in the analyses of all cerebrospinal fluid, and non-specific pleocytosis was observed in three dogs. The two dogs that received chemotherapy were euthanized within 1 month of discharge. Considering the diagnosis of acute leukemia is rarely incidental and, in the absence of other diagnoses, CNS involvement of AL was considered highly likely in the cases described. However, the histopathology for confirmation was not performed.

Neurological signs associated with AL of the CNS have been hypothesized to be due to leptomeningeal infiltration, spinal cord infiltration, vascular events (haemorrhagic or thromboembolic events), or the presence of a compressive mass (24–26). In dogs, neurological signs have been reported in 17–19% of dogs with AL, particularly in acute myeloid leukemia, acute lymphoblastic leukemia, and English bulldog T-cell leukemia (8, 10, 27). Despite the prevalence of neurological signs, only four case reports have confirmed CNS involvement, based on CSF cytology or CNS histopathology. Two of these cases were confirmed during the post-mortem examination (13–16). Neoplastic cells were found infiltrating the cranial nerves, brain, brain and/or spinal cord meninges, spinal cord grey matter, epidural adipose tissue, nerve roots, extradural space, and extra-central nervous system (the lymph nodes, thymus, thyroid, myocardium, pericardium, lungs, liver, spleen, intestines, adrenal glands, kidney, prostate, bone marrow, and focal or diffuse skeletal muscle) (13–16). The leptomeningeal involvement of the brain, also called leukemic meningitis, was suspected to occur via the arachnoid vein and subsequently the CSF (28). In human medicine, CNS involvement is rare and mainly asymptomatic, occurring in less than 3% of children and 5–10% of adults with acute leukemia (28–30). However, one-third of cases relapse in the central nervous system (24, 30).

Due to the absence of circulating blasts or ambiguous cytomorphology (reported in 15% of dogs), peripheral blood cytology is not always diagnostic for AL, as was observed in the cases described (8). The most common haematological and biochemical laboratory abnormalities observed were bicytopenia (thrombocytopenia and/or anemia) and a moderate elevation in ALP, as previously reported (7, 12, 31). Myelophthisis, the downregulation of erythropoiesis, and secondary immune-mediated destruction are the possible causes of the anemia and thrombocytopenia observed in canine leukemia (1). Increased ALP may be due to leakage from blast cell death or the production of isoenzymes, bone marrow remodulation, or secondary hepatic infiltration. However, contrary to other cancer types, it has not been identified as a monitoring tool or prognostic factor (32).

The diagnosis of AL of the CNS in human medicine requires a combination of clinical signs, CSF cytospin, +/− flow cytometry, +/− MRI, and specific cases of meningeal/tissue biopsy (24, 27, 29, 30, 32). A focal or diffuse nodular leptomeningeal enhancement and enlargement of the cranial nerves on MRI are considered indicative of CNS involvement and warrant local treatment (33, 34). Pachymeningeal enhancement at two or more sites in the brain is a negative prognostic factor (28). The diagnostic imaging findings in the current study resemble human literature and Single AL MRI reported. The tentative differential diagnosis of MRI findings included meningitis of unknown aetiology or lymphoma. Myositis was suspected in three dogs based on MRI findings. Only one of the three dogs with muscle changes had a biopsy, and this was consistent with sterile neutrophilic inflammation. The musculoskeletal involvement occurs in 8–38% of human patients with acute leukemia and has been associated with sterile neutrophilic inflammation, polymyositis, dermatomyositis, or secondary infections (35–38). Diffuse skeletal muscle infiltration of neoplastic cells has been a finding in dogs during necropsy (13, 15, 16).

The MRI scan is of particular relevance in patients with AL and normal CSF analysis but with a high clinical suspicion of CNS involvement (33). Historically, the diagnosis of leukemia of the CNS relied mainly on the cytospin of the CSF. However, because of the low sensitivity associated with the diagnosis of leukemia (sensitivity <50%; specificity >95%), the addition of CSF flow cytometry is recommended (sensitivity 78%) (24, 27, 30). The cytology results may be non-representative if only a small number of neoplastic cells are present in a large volume of CSF, particularly in cases with minimal leptomeningeal involvement (29, 30). The increased level of CSF protein is a common finding in humans with AL despite normal cytology analysis. In the study reported here, CSF cytology was not diagnostic for leukemia. The predominance of monocytoid cells and mixed pleocytosis observed in this study are non-specific and can be observed with any cause of CNS necrosis or myelomalacia and have been associated with various disease processes such as granulomatous meningoencephalitis, infectious agents (e.g., fungal, protozoal, or *Ehrlichia*), and steroid-responsive meningitis; however, less frequently with meningioma (39, 40). Flow cytometry was only performed on peripheral blood.

Bone marrow changes on MRI in people with acute leukemia are well documented and may precede changes in laboratory tests and tissue analysis (21). The most common patterns on MRI are diffuse, patchy, and focal abnormal hypointense T1W signals (20). In children, evidence of long bone marrow remodulation on MRI (diffuse replacement pattern) has been consistently associated with leukemia in comparison to lymphoma (20, 21). There are no studies in veterinary medicine assessing bone marrow in cases of hematopoietic neoplasia in dogs. However, in this study, three out of the four dogs with complete MRI studies had bone marrow changes as observed in the sternum, manubrium, and calvarial bones. The dog without bone marrow changes was diagnosed with AL by bone marrow cytology. This subject only had the vertebral bones included in the MRI study, and the vertebral bones have been shown to have less bone marrow (41).

This study has several limitations, including the small population, the retrospective nature of the study, a lack of a consistent MRI protocol, the absence of a necropsy for a definitive diagnosis of leukemia in the CNS, and the need to determine the cause of the MRI findings. Meningoencephalomyelitis, the main differential diagnosis, could not be differentiated based on clinical presentation, MRI, or CSF analysis. However, it was considered less likely due to the cytopenia observed in hematology reports and lymphadenopathy on MRI. MRI changes in dogs with leukemia have only been described in one dog, and no comparisons with other veterinary studies were possible. An additional limitation is that the MRI studies were limited to areas determined by neurological examination changes, and a whole-body MRI could have provided more information. The data search method may have decreased the case numbers. The cases diagnosed by the referring veterinarians with neurological signs may have been referred to other departments and were not included in the study. However, as AL is a rare diagnosis, this may not have hindered the cases.

The diagnosis of leukemia in the CNS in veterinary medicine is challenging due to the low sensitivity of CSF cytology, the infrequent use or validity of CSF flow cytometry in veterinary medicine, and the uncommon practice of biopsying the central nervous system. The MRI findings are well described in humans with AL of the CNS and are considered diagnostic; however, there is no similar evidence in dogs. Most of the cases reported had the diagnosis confirmed during the post-mortem examination. The clinical diagnosis of leukemia in the CNS may therefore depend on a combination of clinical findings and possibly the addition of flow cytometry. Dappiano et al. (16) suggested that acute leukemia should be included as a differential diagnosis in dogs with neurological signs due to spinal cord compression with other findings (pyrexia, lymphadenopathy, hepatomegaly, marked leukocytosis, anemia, and thrombocytopenia). In the current study, dogs had mild or the absence of signs of systemic disease on presentation. As previously discussed, peripheral blood cytology is not always a diagnosis for AL. In addition, some dogs present without peripheral cytopenia (5–10%) (8, 9, 12). This may lead to underdiagnosed CNS neoplasia based on routine testing. Within other differentials, dogs with diffuse T2W hyperintense lesions on the brain or spinal cord, meningeal enhancement, and bone marrow remodeling on MRI may suggest CNS hematopoietic neoplasia. Additional studies are required to evaluate the role of flow cytometry on canine CSF neoplasia, the compatibility of the MRI findings described, and histopathology. The recognition of CNS involvement in human medicine has led to a significant improvement in outcomes (30, 42) mainly due to the use of CNS-targeted therapy (intra-thecal chemotherapy and radiotherapy) in both prophylactic and treatment settings (24, 30, 33, 41).

## 5 Conclusion

The diagnosis of CNS involvement in AL was not confirmed despite advanced investigations in the majority of cases. Further studies are needed to assess whether, as in human medicine, a combination of clinical findings is sufficient to attain a diagnosis and implement local treatment. Furthermore, additional studies are needed to evaluate the role of MRI in detecting hematopoietic disease in veterinary medicine.

## Data availability statement

The original contributions presented in the study are included in the article/supplementary material, further inquiries can be directed to the corresponding author.

## Ethics statement

Ethical approval was approved by Professor David Morton CBE MRCVS Chair of the RCVS Ethics Review Panel. The studies were conducted in accordance with the local legislation and institutional requirements. Written informed consent was not obtained from the owners for the participation of their animals in this study because retrospective nature. There was consent for data usage. Ethical approval was not required for the study involving humans.

## Author contributions

FL: Writing – original draft. CP: Writing – review & editing. RD: Writing – review & editing. GC: Writing – review & editing.
